# Characteristics in childhood and adolescence associated with future multiple sclerosis risk in men: cohort study

**DOI:** 10.1111/ene.12718

**Published:** 2015-04-27

**Authors:** M. Gunnarsson, R. Udumyan, S. Bahmanyar, Y. Nilsagård, S. Montgomery

**Affiliations:** ^1^ Department of Neurology and NeurophysiologyFaculty of Medicine and HealthÖrebro UniversityÖrebroSweden; ^2^ Department of Clinical Epidemiology and BiostatisticsFaculty of Medicine and HealthÖrebro UniversityÖrebroSweden; ^3^ Clinical Epidemiology Unit and Centre for PharmacoepidemiologyDepartment of MedicineKarolinska Institutet, Karolinska HospitalStockholmSweden; ^4^ Faculty of MedicineGolestan University of Medical SciencesGorganIran; ^5^ Faculty of Medicine and HealthMedicineÖrebro UniversityÖrebroSweden; ^6^ Clinical Epidemiology UnitDepartment of MedicineKarolinska University HospitalKarolinska InstitutetStockholmSweden; ^7^ Research Department of Epidemiology and Public HealthUniversity CollegeLondonUK

**Keywords:** cognitive function, cohort, multiple sclerosis, physical fitness, stress

## Abstract

**Background and purpose:**

Associations with multiple sclerosis (MS) of living conditions in childhood and characteristics in adolescence including physical fitness, cognitive function and psychological stress resilience were investigated.

**Methods:**

A cohort of male Swedish residents born 1952–1956 who were included in the Swedish Military Conscription Register was used to create a nested case−control study comprising 628 MS cases and 6187 controls matched on birth year, county of residence and vital status at time of diagnosis. Conscription examination records were linked with other national register data. Conditional logistic regression was used to evaluate associations with MS subsequent to the conscription examination.

**Results and conclusions:**

Men with MS were less likely to be from more crowded households in childhood (>two persons per room) with an adjusted odds ratio of 0.67 (95% confidence interval 0.51–0.86, *P* = 0.023). They had lower physical working capacity in adolescence with adjusted odds ratio of 0.94 (95% confidence interval 0.89–0.99, *P* = 0.026). Cognitive function and stress resilience scores displayed no significant differences between cases and controls. Parental occupation in childhood and body mass index in adolescence were not associated with future MS risk. The inverse association of MS risk with higher levels of household crowding may reflect environmental factors such as the pattern of exposure to microorganisms. Lower physical fitness in men at MS risk may indicate a protective effect of exercise or could be due to prodromal disease activity, although there was no association with cognitive function. Poor psychological stress resilience (and thus risk of chronic stress arousal) was not associated with MS.

## Introduction

Multiple sclerosis (MS) is an immune‐mediated disease with typical onset in young adults. A variety of genes have been associated with MS risk, but associations with environmental exposures indicate the importance of gene−environment interactions 1, 2, 3. It is well established that MS prevalence increases north and south from the equator 4 and migrants from a high to a low incidence area retain their native risk only if migration occurs after 15 years of age. These findings suggest that environmental factors in childhood or adolescence are important in determining lifetime MS risk 4.

There is evidence that the pattern of pre‐adulthood exposure to infectious agents is implicated in MS aetiology. An atypical pattern of Epstein–Barr virus (EBV) infection is overrepresented in MS 5 and antibody reactivity against EBV nuclear antigen‐1 seems to be associated with increased MS risk, suggesting an altered immune response to the virus in susceptible individuals 6. Interaction with 25‐hydroxyvitamin D status and EBV infection has recently been proposed to influence the immune response and modulate MS risk 7.

Lifestyle factors such as smoking and obesity have also been associated with increased risk of developing MS 8, 9, 10, 11, whilst alcohol has been associated with reduced MS risk 12, but not in all studies 13. Findings on parental socioeconomic circumstances and stressful life events during childhood are inconsistent 14, 15, 16, 17, 18 but some studies may have been limited by a lack of prospectively collected information.

Here prospectively recorded Swedish register data were used to examine associations of familial and personal characteristics in childhood and adolescence with adult‐onset MS amongst a representative sample of men in a nested case–control study. Childhood circumstances were captured through information on parental occupation and household crowding, which is relevant to a variety of exposures, including pattern of exposure to microorganisms. A compulsory military conscription examination in late adolescence provided several measures. Stress resilience, indicating susceptibility to psychological stress, was assessed through interview by a psychologist and has previously been used to assess the role of stress in the aetiology of other diseases 19. MS may have a long and silent natural history, so damage to the central nervous system may occur prior to diagnosis. Therefore, putative pre‐diagnostic influence on cognitive and physical function was examined. Exercise may, however, also protect from inflammatory disease 20. Thus, physical working capacity and body mass index (BMI) were included in the study.

## Methods

The study took its subjects from a cohort of 284 198 men born between 1 January 1952 and 31 December 1956 that were included in the Swedish Military Conscription Register. Men in this cohort had their conscription examinations in the 1970s, except for 80 men in 1969 and 1163 men in the 1980s. Examinations were performed at ages 18 and 19 years, with a small number at later ages. At that time, assessment for military service was compulsory in Sweden. Fewer than 4% of men were excluded from the enlistment examination due to chronic illness or disability.

In the cohort of 284 198 men, 2564 were excluded due to data inconsistencies such as errors in the personal identification number, female sex or uncertain vital status. Further, 182 men were excluded due to improbable measures at the conscription assessment: height <144 cm (*n* = 39), BMI below 15 (*n* = 134) and weight above 178 kg (*n* = 9). Individuals with MS, optic neuritis or demyelinating disease at the time of conscription examination in adolescence were also excluded as the study assessed MS risk after this time. The sample eligible for inclusion after the conscription examination date comprised 281 268 men. Next, men with missing data for covariates used in the analysis (*n* = 36 311) and potential controls with diagnoses of optic neuritis (ICD‐8, 367; ICD‐9, 377D, 377.3; ICD‐10, H46) or demyelinating disease (ICD‐8, 341; ICD‐9, 341; ICD‐10, G36, G37) (*n* = 200) were excluded. The sample available for generating a nested case–control study comprised 244 757 men.

Approximately 4% of the potential subjects did not attend the assessments due to chronic illness, disability or because they were not Swedish citizens. Amongst the remaining men with missing data approximately 76% had health conditions that resulted in their being classified as medically or intellectually unfit for military service, and therefore they did not undergo some further conscription examinations resulting in missing data. Thus, the cohort is somewhat selected for better health in adolescence.

### Multiple sclerosis cases and matched controls

Multiple sclerosis was defined using ICD‐8, 340; ICD‐9, 340; ICD‐10, G35 codes from the Swedish National Patient Register (NPR), with information on inpatient and outpatient diagnoses 21, 22. The register attained full coverage in 1987 and outpatients were added in 2001. The analysis is based on both primary (*n* = 572) and secondary (*n* = 56) incident MS diagnoses (cases, *n* = 628) identified through prospectively recorded data from the NPR within a time window that spanned from the date of conscription examination until 31 December 2009.

For the analysis, a nested case–control study data set was generated from the cohort. For each case (a man with an MS diagnosis), 10 controls (in some risk sets fewer than 10 controls were identified) were selected at random amongst members of the cohort who were alive, resident in Sweden and without MS diagnosis. The controls were matched individually with cases by vital status at MS diagnosis (controls were alive and living in Sweden), as well as birth year and county of residence in 1970. The county of residence measure comes from the 1970 population census as the classification corresponds to the administrative boundaries used to determine which specific disease coding system is used (these varied over time).

## Measures

### Swedish Military Conscription Register

The Swedish Military Conscription Register 23 records extensive and standardized physical and psychological examinations by physicians and psychologists. Measures included height (cm) and BMI (kg/m^2^). A physical working capacity score was assessed using a cycle ergonomic test performed after evaluation of the resting electrocardiogram and medical history. A 5‐min submaximal test was directly followed by a maximal test with gradually increasing load until exhaustion. The results were transformed into scores from 0 (lowest) to 9 (highest). A stress resilience score was determined from a psychological examination that assessed the potential ability to cope with stress, based on the ability to control and channel nervousness, tolerance of stress and disposition to anxiety, based on reported prior experiences in everyday life 24. The scores were grouped into low (1–3), medium (4–6) and high (7–9) stress resilience. A cognitive function score comprised four subtests, linguistic understanding, spatial recognition, general knowledge and ability to follow mechanical instructions, and was transformed into a single score (1–9). A summary disease score (0–9) denotes the presence and severity of disease relevant to the capability to undertake military duty. The scores were classified into five groups ranging from 0 (very significant problem) to 9 (no diagnosis).

Erythrocyte sedimentation rate (ESR) and erythrocyte volume fraction were recorded to assess systemic inflammation.

### Socioeconomic and demographic characteristics

Demographic data, including information on vital status and emigration, were provided by the government organization Statistics Sweden using the Total Population Register 25. Markers of material and social circumstances in childhood were taken from the 1960 census. Household crowding during childhood was calculated (people per habitable room) and transformed into quarters of its distribution. Number of siblings of the study participants was recorded. Occupation of the head of household was classified as manual, agricultural, farm owners/managers, office workers, business owners/managers and others.

### Statistical analysis

Descriptive statistics included frequencies, proportions, means and median values. Conditional logistic regression was used to examine unadjusted and adjusted associations between MS with onset after the conscription examination with characteristics in childhood and adolescence. The matched analysis took into account birth year and the 25 counties of residence, as well as the age at MS diagnosis. The main model included the following measures: household crowding, parental occupation, BMI, summary disease score (modelled as categorical variables); and height, stress resilience score, physical working capacity and cognitive function score (modelled as continuous variables). The functional form of the measures was explored using multivariable fractional polynomial modelling 26. This analysis indicated a linear relationship with the log odds of the association with the outcome for height, stress resilience score, cognitive function score and physical working capacity score, and these were therefore modelled continuously.

In the initial analysis both number of siblings and household crowding were included in the same model, but due to collinearity between these measures the number of siblings was dropped as household crowding explained more of the variance. ESR (as a marker of systemic inflammation) adjusted for erythrocyte volume fraction was also included in the models, but due to a lack of association with MS and no notable influence on the other associations it was not included in the final model. A sensitivity analysis was performed by excluding cases with MS (and their matched controls) diagnosed within 5 years from the conscription examination to reduce the possibility that associations are being driven entirely by MS with onset soon after the conscription examination.

The statistical software used was Stata version 12/SE for Windows (StataCorp, College Station, TX, USA). Tests were two‐sided and statistical significance was defined as *P* < 0.05 and 95% confidence intervals (95% CI) that do not include 1.00.

## Results

The characteristics of the study population comprising 628 men with MS (cases) and 6187 men without MS (controls) are described in Table 1. Cases tended to come from less crowded households and were slightly taller. MS cases had a lower physical working capacity score and were more likely to have a summary disease score indicating the presence of other diagnoses.

**Table 1:** (a) Characteristics of the cases and controls, categorical variables. (b) Characteristics of the cases and controls, continuous variables

	Controls	Cases	*P* value
	*N* = 6187	*N* = 628	
(a)			
Household crowding^a^ (persons/room) 1960 census			
0.2–1.25	1532 (24.8)	189 (30.1)	0.007^b^
1.29–1.67	1917 (31.0)	195 (31.1)	
1.71–2	1372 (22.2)	134 (21.3)	
2.14–13	1366 (22.1)	110 (17.5)	
Head of household's occupation 1960 census			
Manual worker	2592 (41.9)	243 (38.7)	0.438^b^
Agricultural worker	235 (3.8)	23 (3.7)	
Farm owner/manager	619 (10.0)	72 (11.5)	
Office worker	1690 (27.3)	190 (30.3)	
Business owner/manager	696 (11.2)	67 (10.7)	
Other	355 (5.7)	33 (5.3)	
BMI categories (kg/m^2^)			
Underweight (<18.5)	732 (11.8)	72 (11.5)	0.967^b^
Normal weight (>18.5, <25.0)	5022 (81.2)	509 (81.1)	
Overweight (>25.0, <30.0)	376 (6.1)	41 (6.5)	
Obese (>30.0)	57 (0.9)	6 (1.0)	
Summary disease score			
Very significant problem (0)	325 (5.3)	31 (4.9)	0.019^b^
Significant problem (2–3)	194 (3.1)	32 (5.1)	
Fairly significant problem (4–5)	468 (7.6)	62 (9.9)	
No serious problem (6–8)	2515 (40.6)	248 (39.5)	
No diagnosis (9)	2685 (43.4)	255 (40.6)	
Height quintiles (cm)			
144–173	1274 (20.6)	117 (18.6)	0.688^b^
174–177	1370 (22.1)	137 (21.8)	
178–180	1122 (18.1)	111 (17.7)	
181–184	1244 (20.1)	135 (21.5)	
185–210	1177 (19.0)	128 (20.4)	
Stress resilience score			
Low (1–3)	1366 (22.1)	144 (22.9)	0.528^b^
Moderate (4–6)	3389 (54.8)	351 (55.9)	
High (7–9)	1432 (23.1)	133 (21.2)	
Cognitive function score			
Low (1–3)	1245 (20.1)	113 (18.0)	0.404^b^
Moderate (4–6)	3312 (53.5)	350 (55.7)	
High (7–9)	1630 (26.3)	165 (26.3)	
Physical working capacity score			
Low (0–3)	347 (5.6)	36 (5.7)	0.164^b^
Moderate (4–6)	3086 (49.9)	337 (53.7)	
High (7–9)	2754 (44.5)	255 (40.6)	

(b)					
Height (cm)	178.7	6.44	179.3	6.40	0.045^c^
Stress resilience score	5.02	1.89	4.93	1.85	0.256^c^
Cognitive function score	5.20	1.96	5.25	1.92	0.505^c^
Physical working capacity score	6.29	1.80	6.16	1.73	0.078^c^

Values given as number (per cent) in (a) and as mean and SD in (b). ^a^Quarters of its distribution produced non‐contiguous ranges for crowding; ^b^ *P* value from chi square test; ^c^ *P* value from *t* test.

In all analyses, the matched design takes into account year of birth, region of residence and age at MS diagnosis. Table 2 shows that lower household crowding in childhood (at ages 4–8 years) has a dose‐dependent association with MS risk, such that the greater the crowding, the lower the risk of MS. Taller stature in adolescence is significantly associated with a higher risk of MS and there was a less notable association with raised MS risk for lower physical working capacity scores. Associations with the summary disease score indicate that MS cases were more likely to have other diagnoses in adolescence. The above associations remained statistically significant in the adjusted model, except for the summary disease score which had marginal significance overall and was significant only for the ‘significant problem’ category. BMI (at ages 18–19 years) as a categorical variable was not statistically significantly associated with MS. In a sub‐analysis, BMI was modelled as a continuous variable and there was a modest association with MS in the adjusted (but not unadjusted) analysis, producing an adjusted odds ratio of 1.03 (95% CI 1.00–1.06). No notable associations were observed for cognitive function and stress resilience score in adolescence or head of household's occupation in childhood.

**Table 2:** Associations with multiple sclerosis

	Unadjusted^a^ OR (95% CI)	*P* value^b^	Adjusted^a^^,^ c OR (95% CI)	*P* value^b^
	*N* = 6815		*N* = 6815	
Household crowding (persons/room), 1960 census				
0.2–1.25	Reference	0.0078	Reference	0.023
1.29–1.67	0.83 (0.67–1.02)		0.84 (0.68–1.04)	
1.71–2	0.79 (0.63–1.00)		0.80 (0.63–1.02)	
2.14–13	0.65 (0.51–0.84)		0.67 (0.51–0.86)	
Head of household's occupation, 1960 census				
Manual worker	Reference	0.431	Reference	0.793
Agricultural worker	1.05 (0.67–1.64)		1.04 (0.66–1.63)	
Farm owner/manager	1.24 (0.94–1.64)		1.17 (0.88–1.55)	
Office worker	1.20 (0.98–1.48)		1.11 (0.89–1.38)	
Business owner/manager	1.03 (0.78–1.37)		0.94 (0.70–1.27)	
Other	1.00 (0.68–1.46)		0.99 (0.68–1.45)	
Height (cm)	1.01 (1.00–1.03)	0.041	1.02 (1.00–1.03)	0.019
BMI categories (kg/m^2^)				
Underweight (<18.5)	0.97 (0.75–1.25)	0.884	0.86 (0.66–1.13)	0.410
Normal weight (≥18.5, <25)	Reference		Reference	
Overweight, obese (≥25)	1.07 (0.78–1.46)		1.13 (0.82–1.55)	
Stress resilience score	0.98 (0.93–1.02)	0.263	0.98 (0.93–1.04)	0.582
Cognitive function score	1.01 (0.97–1.06)	0.517	1.01 (0.96–1.06)	0.706
Physical working capacity score	0.96 (0.92–1.01)	0.085	0.94 (0.89–0.99)	0.026
Summary disease score				
Very significant problem (0)	1.00 (0.68–1.49)	0.021	0.96 (0.62–1.47)	0.046
Significant problem (2–3)	1.73 (1.17–2.57)		1.67 (1.10–2.53)	
Fairly significant problem (4–5)	1.40 (1.04–1.88)		1.34 (0.99–1.81)	
No serious problem (6–8)	1.04 (0.86–1.25)		1.02 (0.84–1.23)	
No diagnosis (9)	Reference		Reference	

^a^The matched analysis took birth year, county of residence and duration of follow‐up (age at MS onset) into account; ^b^ *P* values are from the Wald tests for the overall association of the variables with MS; ^c^adjusted for all variables shown.

When ESR was examined with adjustment only for erythrocyte volume fraction, or in the multivariate model, no statistically significant associations were observed and its inclusion did not alter any of the other associations at the level of precision reported in the tables. Compared with 1 mm, the adjusted odds ratios were 0.86 (95% CI 0.70–1.06), 1.06 (95% CI 0.73–1.53) and 1.31 (95% CI 0.83–2.05) for 2–6 mm, 7–10 mm or over 10 mm, respectively. In *post hoc* analyses specific diseases that explain the association of the summary disease score with MS risk could not be identified. The broad category containing inflammatory diseases of the skin and connective tissue was somewhat overrepresented in cases (data not shown), but no firm conclusions could be drawn. The exclusion of MS diagnoses in the 5 years following the conscription examination did not alter any of the results notably (data not shown).

To assess potential selection bias due to missing values from the conscription assessment, the association of household crowding in childhood with MS risk was examined amongst all available subjects. The associations were virtually unaltered (data not shown) suggesting almost no selection effect of missing values for this finding.

## Discussion

The present study shows that individuals living in more crowded households during childhood (at ages 4–8 years) were less likely to develop MS. No evidence was found of associations with impaired cognitive function or lower stress resilience in late adolescence, but cases already had somewhat lower physical fitness in adolescence, prior to their MS diagnosis. The association of higher BMI in adolescence with MS was equivocal. ESR in adolescence, a marker of systemic inflammation, was not associated with MS.

It is likely that household crowding alters the pattern of environmental exposures, including to microorganisms. During childhood such conditions may influence the nascent immune system through, for example, gut colonization, infection and thus acquired immunity. There is evidence implicating infectious agents in the aetiology of MS 4. The role of EBV has been investigated and debated extensively. MS patients tend to be infected at a later age 27, 28, 29, suggesting that a primary EBV infection in earlier childhood may be less of a risk than infectious mononucleosis in adolescence. It has been proposed that an altered immune response to EBV following late infection in susceptible individuals could be an important MS risk 6. In our study, a dose‐dependent association of greater household crowding when the subjects were 4–8 years of age with decreased risk of MS was found. It is hypothesized that household crowding is a marker of increased exposure to infectious agents in childhood, such that infections like EBV may occur at an earlier age. An alternative explanation for the inverse association between greater household crowding and MS risk is reduced parental fertility in MS 30. However, this is unlikely as household crowding was a better predictor of MS than number of siblings. Crowding may be the consequence of greater financial adversity and poorer socioeconomic circumstances have in fact been associated with lower MS risk 31. In our study, the association of crowding was independent of parental occupation, a marker of material and cultural circumstances, which itself was not associated with MS.

It is hypothesized that exposures similar to those associated with household crowding explain the findings for stature; for example, sharing a bedroom (which will not always be captured by the crowding variable) may result in disturbed sleep and slowed growth, whilst also altering patterns of exposure to microorganisms. Greater household crowding has been associated with reduced physical growth 32. Growth hormone and other factors relevant to development in childhood are secreted during deep sleep 33, 34, and frequently disrupted sleep in more crowded households is a putative explanation.

The association of lower physical fitness in late adolescence (ages 18–19 years) with higher subsequent MS risk can have several possible explanations. One possibility is due to prodromal tissue damage in the central nervous system. However, no evidence of pre‐diagnostic impaired cognitive function was found. Exercise has been suggested to protect against other inflammatory diseases 20, which may be an alternative interpretation of our findings for physical working capacity. The association of BMI at ages 18–19 years with MS risk was equivocal. Earlier studies have identified a raised risk of MS in those with higher BMI 10, 35. At the time of the conscription examinations, obesity in adolescence was still relatively rare in Sweden and may help explain the lack of association with our categorical measure of BMI. The weak association with the continuous measure is consistent with some association of BMI with MS risk. It is noteworthy that higher BMI cannot explain the association of MS with lower physical working capacity, as demonstrated by the adjusted analysis. The lack of association with stress resilience suggests that psychological stress is unlikely to be particularly important in causing MS. The objectively assessed measure of stress resilience has been shown to predict risk of other diseases such as stroke 19, as low resilience makes individuals more susceptible to stressful exposures in daily life. This measure benefits from being recorded prior to MS onset and is unlikely to be influenced by differential reporting bias.

A strength of our study is the collection of information prior to MS diagnosis (clinical onset of MS tends to occur between 20 and 40 years of age and those with MS at the conscription examination were excluded). In a sub‐analysis, individuals with an MS diagnosis in the 5 years following the conscription examination were excluded and results were not notably altered. A disadvantage is that women, who make up the majority of MS patients, could not be included as military conscription was almost exclusively limited to males in the study period. Diagnoses from the NPR may not always be accurate, but in a previous study similar results for MS patients from this register and those whose diagnosis could be confirmed in the Swedish MS register were found 36. MS diagnosis has not been specifically validated in the NPR but general estimates suggest acceptably high diagnostic accuracy 22. Since outpatients were added to the NPR in 2001, patients with a more benign disease course treated only as outpatients before 2001 and not subsequently may not have been included in our study. However, based on our experience of the Swedish MS register (not used in this study), it is reasonable to believe that this is a limited number of MS cases. Other potential limitations include lack of information on other factors relevant to MS risk such as cigarette smoking 9, 11. Although not a substitute for detailed information on smoking, adjustment was made for ESR indicating systemic inflammation which has been related to heavy smoking, although not always consistently 37. The study was not designed to identify the specific diagnoses that might account for the weak association between the summary disease score in adolescence and subsequent MS, but adjustment for this score is useful as it shows the associations with MS are independent of other diseases already present at the conscription examination.

In conclusion, it is suggested that the inverse association with MS for household crowding at ages 4–8 years is explained by patterns of infectious exposure in childhood relevant to future MS risk. There was no evidence that chronic psychosocial stress, indicated by stress resilience, is implicated in the aetiology of MS. Early adverse consequences of disease processes prior to MS diagnosis could not be seen for cognitive function in adolescence. Associations with physical capacity may reflect a reduced ability to exercise amongst those who would develop MS or there may be a protective effect of physical fitness.

## Disclosure of conflicts of interest

The authors declare no financial or other conflicts of interest.

## Ethics approval

This study was conducted with the approval of the Regional Ethics Committee in Uppsala, Sweden (Dnr 2009/306).

## References

[1] De Jager PL , Simon KC , Munger KL , *et al* Integrating risk factors: HLA‐DRB1*1501 and Epstein–Barr virus in multiple sclerosis. Neurology 2008; 70: 1113–1118. 18272866 10.1212/01.wnl.0000294325.63006.f8

[2] Sawcer S , Franklin RJ , Ban M . Multiple sclerosis genetics. Lancet Neurol 2014; 13: 700–709. 24852507 10.1016/S1474-4422(14)70041-9

[3] Sundstrom P , Nystrom M , Ruuth K , *et al* Antibodies to specific EBNA‐1 domains and HLA DRB1*1501 interact as risk factors for multiple sclerosis. J Neuroimmunol 2009; 215: 102–107. 19733917 10.1016/j.jneuroim.2009.08.004

[4] Kurtzke JF . Multiple sclerosis in time and space–geographic clues to cause. J Neurovirol 2000; 6(Suppl. 2): S134–S140. 10871801

[5] Pakpoor J , Disanto G , Gerber JE , *et al* The risk of developing multiple sclerosis in individuals seronegative for Epstein–Barr virus: a meta‐analysis. Mult Scler 2013; 19: 162–166. 22740437 10.1177/1352458512449682

[6] Sundstrom P , Juto P , Wadell G , *et al* An altered immune response to Epstein–Barr virus in multiple sclerosis: a prospective study. Neurology 2004; 62: 2277–2282. 15210894 10.1212/01.wnl.0000130496.51156.d7

[7] Salzer J , Nystrom M , Hallmans G , *et al* Epstein–Barr virus antibodies and vitamin D in prospective multiple sclerosis biobank samples. Mult Scler 2013; 19: 1587–1591. 23549431 10.1177/1352458513483888

[8] Munger KL , Chitnis T , Ascherio A . Body size and risk of MS in two cohorts of US women. Neurology 2009; 73: 1543–1550. 19901245 10.1212/WNL.0b013e3181c0d6e0 PMC2777074

[9] Hedstrom AK , Baarnhielm M , Olsson T , *et al* Tobacco smoking, but not Swedish snuff use, increases the risk of multiple sclerosis. Neurology 2009; 73: 696–701. 19720976 10.1212/WNL.0b013e3181b59c40

[10] Hedstrom AK , Olsson T , Alfredsson L . High body mass index before age 20 is associated with increased risk for multiple sclerosis in both men and women. Mult Scler 2012; 18: 1334–1336. 22328681 10.1177/1352458512436596

[11] Riise T , Nortvedt MW , Ascherio A . Smoking is a risk factor for multiple sclerosis. Neurology 2003; 61: 1122–1124. 14581676 10.1212/01.wnl.0000081305.66687.d2

[12] Hedstrom AK , Hillert J , Olsson T , *et al* Alcohol as a modifiable lifestyle factor affecting multiple sclerosis risk. JAMA Neurol 2014; 71: 300–305. 24395432 10.1001/jamaneurol.2013.5858

[13] Massa J , O'Reilly EJ , Munger KL , *et al* Caffeine and alcohol intakes have no association with risk of multiple sclerosis. Mult Scler 2013; 19: 53–58. 22641303 10.1177/1352458512448108 PMC3434262

[14] Briggs FB , Acuna BS , Shen L , *et al* Adverse socioeconomic position during the life course is associated with multiple sclerosis. J Epidemiol Community Health 2014; 68: 622–629. 24577137 10.1136/jech-2013-203184

[15] Nielsen NM , Jorgensen KT , Bager P , *et al* Socioeconomic factors in childhood and the risk of multiple sclerosis. Am J Epidemiol 2013; 177: 1289–1295. 23660795 10.1093/aje/kws350

[16] Nielsen NM , Pedersen BV , Stenager E , *et al* Stressful life‐events in childhood and risk of multiple sclerosis: a Danish nationwide cohort study. Mult Scler 2014; 20: 1609–1615. 24687805 10.1177/1352458514528761

[17] Riise T , Mohr DC , Munger KL , *et al* Stress and the risk of multiple sclerosis. Neurology 2011; 76: 1866–1871. 21624985 10.1212/WNL.0b013e31821d74c5 PMC3115807

[18] Spitzer C , Bouchain M , Winkler LY , *et al* Childhood trauma in multiple sclerosis: a case–control study. Psychosom Med 2012; 74: 312–318. 22408134 10.1097/PSY.0b013e31824c2013

[19] Bergh C , Udumyan R , Fall K , *et al* Stress resilience in male adolescents and subsequent stroke risk: cohort study. J Neurol Neurosurg Psychiatry 2014; 85: 1331–1336. 24681701 10.1136/jnnp-2013-307485 PMC4251543

[20] Khalili H , Ananthakrishnan AN , Konijeti GG , *et al* Physical activity and risk of inflammatory bowel disease: prospective study from the Nurses’ Health Study cohorts. BMJ 2013; 347: f6633. 24231178 10.1136/bmj.f6633 PMC3935281

[21] The National Patient Register . Available from: http://www.socialstyrelsen.se/halsodataregister/patientregistret (accessed 13/05/2014).

[22] Ludvigsson JF , Andersson E , Ekbom A , *et al* External review and validation of the Swedish national inpatient register. BMC Public Health 2011; 11: 450. 21658213 10.1186/1471-2458-11-450 PMC3142234

[23] Whitley E , Batty GD , Gale CR , *et al* Intelligence in early adulthood and subsequent risk of unintentional injury over two decades: cohort study of 1 109 475 Swedish men. J Epidemiol Community Health 2010; 64: 419–425. 19955099 10.1136/jech.2009.100669 PMC4170759

[24] Nilsson PM , Nyberg P , Ostergren PO . Increased susceptibility to stress at a psychological assessment of stress tolerance is associated with impaired fetal growth. Int J Epidemiol 2001; 30: 75–80. 11171861 10.1093/ije/30.1.75

[25] Statistics Sweden. Available from: http://www.scb.se/sv_/Vara-tjanster/SCBs-data-for-forskning/SCBs-datalager/ (accessed 13/05/2014).

[26] Sauerbrei W , Royston P . Building multivariable prognostic and diagnostic models: transformation of the predictors using fractional polynomials. Corrigendum: 2002; 165: 399–400. J Roy Stat Soc 1999; 4 Series A: 9.

[27] Haahr S , Koch‐Henriksen N , Moller‐Larsen A , *et al* Increased risk of multiple sclerosis after late Epstein–Barr virus infection: a historical prospective study. Mult Scler 1995; 1: 73–77. 9345455 10.1177/135245859500100203

[28] Hernan MA , Zhang SM , Lipworth L , *et al* Multiple sclerosis and age at infection with common viruses. Epidemiology 2001; 12: 301–306. 11337603 10.1097/00001648-200105000-00009

[29] Marrie RA , Wolfson C , Sturkenboom MC , *et al* Multiple sclerosis and antecedent infections: a case–control study. Neurology 2000; 54: 2307–2310. 10881258 10.1212/wnl.54.12.2307

[30] Montgomery SM , Lambe M , Olsson T , *et al* Parental age, family size, and risk of multiple sclerosis. Epidemiology 2004; 15: 717–723. 15475721 10.1097/01.ede.0000142138.46167.69

[31] Visser EM , Wilde K , Wilson JF , *et al* A new prevalence study of multiple sclerosis in Orkney, Shetland and Aberdeen city. J Neurol Neurosurg Psychiatry 2012; 83: 719–724. 22577232 10.1136/jnnp-2011-301546

[32] Christiansen N , Mora JO , Herrera MG . Family social characteristics related to physical growth of young children. Br J Prev Soc Med 1975; 29: 121–130. 1182354 10.1136/jech.29.2.121 PMC478901

[33] Preece M . Prepubertal and pubertal endocrinology In: Falkner J, Tanner J, eds. Human Growth, 2nd edn London: Plenum Press, 1985.

[34] Preece M , Holder A . The somatomedins; a family of serum growth factors In: O'Riordan J, ed. Recent Advances in Endocrinology and Metabolism. Edinburgh: Churchill Livingstone, 1982.

[35] Munger KL , Bentzen J , Laursen B , *et al* Childhood body mass index and multiple sclerosis risk: a long‐term cohort study. Mult Scler 2013; 19: 1323–1329. 23549432 10.1177/1352458513483889 PMC4418015

[36] Bahmanyar S , Montgomery SM , Hillert J , *et al* Cancer risk among patients with multiple sclerosis and their parents. Neurology 2009; 72: 1170–1177. 19332695 10.1212/01.wnl.0000345366.10455.62

[37] Pincherle G , Shanks J . Value of the erythrocyte sedimentation rate as a screening test. Br J Prev Soc Med 1967; 21: 133–136. 6033484 10.1136/jech.21.3.133 PMC1059086

